# B3GNT3 overexpression promotes tumor progression and inhibits infiltration of CD8^+^ T cells in pancreatic cancer

**DOI:** 10.18632/aging.202255

**Published:** 2020-12-09

**Authors:** Hongkai Zhuang, Zixuan Zhou, Zedan Zhang, Xinming Chen, Zuyi Ma, Shanzhou Huang, Yuanfeng Gong, Chuanzhao Zhang, Baohua Hou

**Affiliations:** 1 Department of General Surgery, Guangdong Provincial People’s Hospital, Guangdong Academy of Medical Sciences, School of Medicine, South China University of Technology, Guangzhou 510080, China; 2 Shantou University of Medical College, Shantou 515000, China; 3 Department of Hepatobiliary Surgery, Shenshan Central Hospital, Sun Yat-Sen Memorial Hospital, Sun Yat-Sen University, Shanwei 516600, China; 4 The Second School of Clinical Medicine, Southern Medical University, Guangzhou 510280, China

**Keywords:** *B3GNT3*, pancreatic cancer, TCGA, tumor-infiltrating lymphocytes, CD8^+^ T cells

## Abstract

Beta-1,3-*N*-acetylglucosaminyltransferase 3 (*B3GNT3*) has been associated with tumor progression in several solid tumors, and inhibits CD8^+^ T cell-mediated anti-tumor immunity in breast cancer. However, little is known about the potential functions of *B3GNT3* in immunosuppression in pancreatic cancer (PC). This study on *B3GNT3* aims to provide novel insights into the mechanisms of immune suppression or evasion in PC. To this end, the clinical significance and oncologic roles of *B3GNT3* were investigated through bioinformatic analysis and *in vitro* studies. Potential associations between the expression of *B3GNT3* and tumor immunity were mainly analyzed by single-sample gene set enrichment analysis (ssGSEA) and immunofluorescence in tissue microarray (TMA). *B3GNT3* overexpression was observed in PC tissue and was associated with larger tumor sizes, higher histologic grades, and poorer overall survival (OS). *B3GNT3* overexpression was associated with the mutation status and expression of driver genes, especially for *KRAS* and *SMAD4*. *B3GNT3* knockdown inhibited the proliferation, invasion, and epithelial-mesenchymal transition (EMT) of PC cells. *B3GNT3* overexpression significantly correlated with decreased infiltration of tumor infiltrating lymphocytes (TILs), especially CD8^+^ T cells. Overall, our results indicate that *B3GTN3* plays a novel role in tumor progression and immunosuppression, thus serving as a potential therapeutic target in PC.

## INTRODUCTION

As one of the most deadly malignancies, pancreatic cancer (PC) caused 4.5% of all deaths related to cancers worldwide, with poor 5-year survival rate of 2-9% [1, 2]. Recent years have affirmed a significant advancement of immune-based therapies in various types of solid tumors, such as hepatocellular carcinoma, melanoma, head and neck cancer, and non-small-cell lung cancer [3–8]. However, so far, these drugs exhibit limited efficacy for advanced PC, because of the low CD8^+^ T cells infiltration within the tumor microenvironment (TME) [9–11]. Therefore, understanding the molecular mechanisms involved in PC immune suppression is fundamental to the development of more effective immune-based therapeutics to improve the clinical outcome of PC.

Beta-1,3-*N*-acetylglucosaminyltransferase 3 (*B3GNT3*) is a protein coding gene that encodes a member of the beta-1,3-*N*-acetylglucosaminyltransferase family [12]. Previous studies demonstrated that *B3GNT3* participates in the development and progression of human malignancies, such as pancreatic cancer, breast cancer, cervical cancer, lung cancer, and non-Hodgkin lymphoma [12–16]. For example, elevated *B3GNT3* expression levels in pancreatic cancer stem cells (PCSCs) regulate stemness by modulating PCSC markers and promoting tumor progression [13]. *B3GNT3* overexpression is also correlated with unfavorable prognosis of patients with non-small cell lung cancer [15]. Moreover, it was reported that *B3GNT3* downregulation enhances the anti-tumor immunity of cytotoxic T cells in triple-negative breast cancer [16]. However, the potential role of *B3GTN3* in the immune suppression of PC has not been explored.

In the current study, we comprehensively investigated the expression of *B3GNT3*, its clinical significance, and its potential biological function in PC using the Gene Expression Omnibus (GEO) and The Cancer Genome Atlas (TCGA). The oncologic role of *B3GNT3* in PC was determined using *in vitro* studies. For the first time, the potential association between *B3GNT3* expression and CD8^+^ T cell infiltration in PC was evaluated using the ESTIMATE algorithm, CIBERSORT algorithm, single sample Gene Set Enrichment Analysis (ssGSEA), and multi-color immunofluorescence in TMA [17–19].

## RESULTS

### *B3GNT3* overexpression predicts poor prognosis in PC

**Figure 1:**
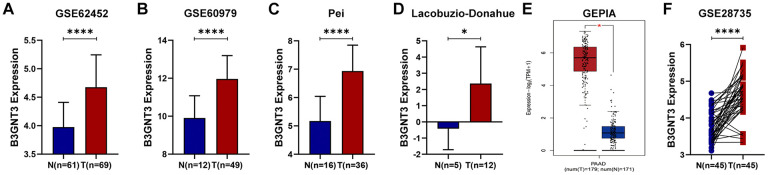
**Multiple databases demonstrated that B3GNT3 was overexpressed in PC.** (**A**–**B**) B3GNT3 expression in GSE62452 and GSE60979 datasets. (**C**–**D**) B3GNT3 expression in the Oncomine database. (**E**) B3GNT3 expression in GEPIA database. (**F**) B3GNT3 expression in GSE28735 dataset. PC, pancreatic cancer; N: normal; T: tumor; GEPIA: Gene Expression Profiling Interactive Analysis. (*P value < 0.05; **P value < 0.01;***P value < 0.001; ****P value < 0.0001).

**Figure 2:**
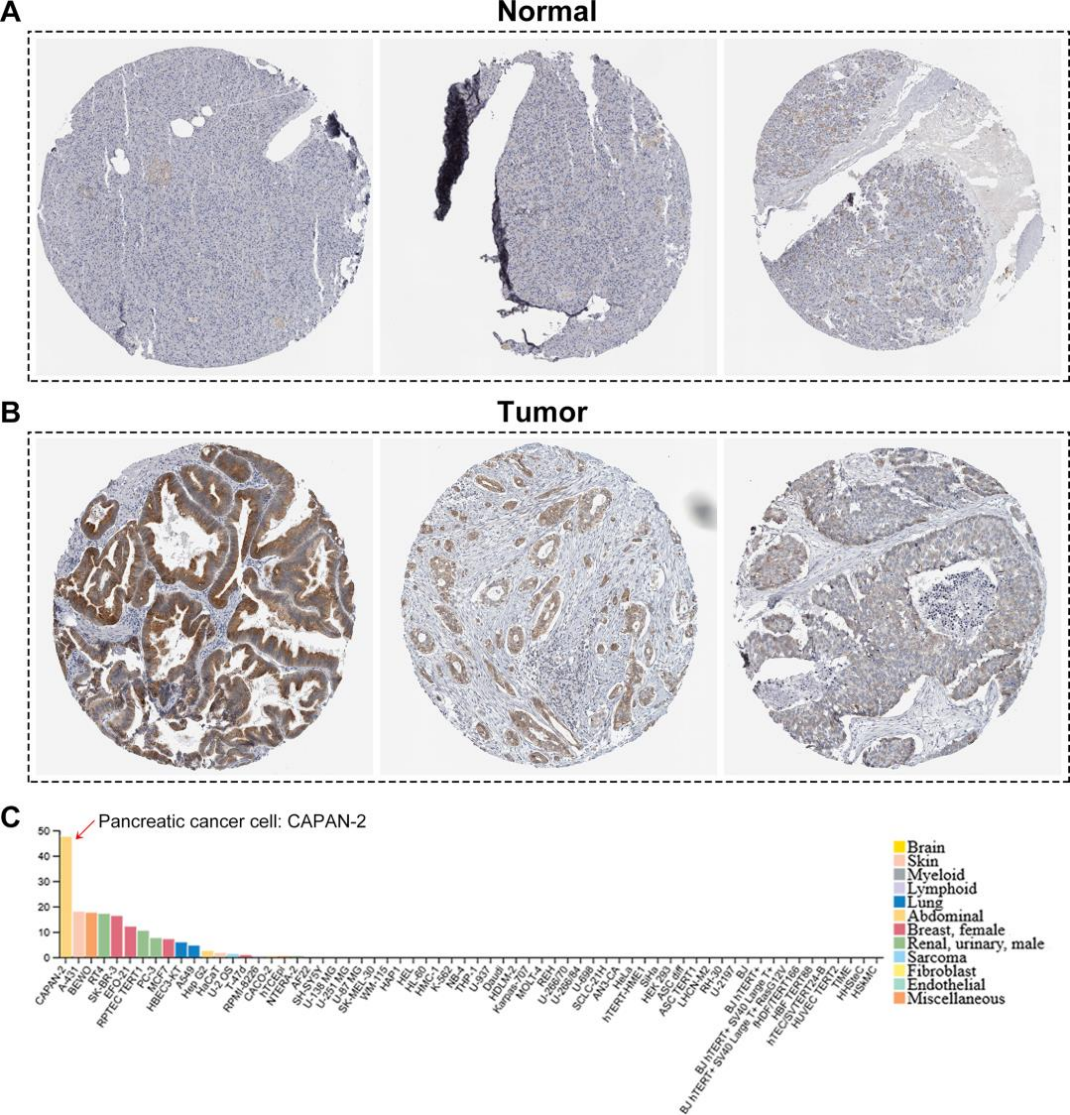
**The human protein atlas database analysis.** (**A**, **B**) The translational differences of *B3GNT3* between pancreatic cancer tissues and normal pancreatic tissues. (**C**) The expression of *B3GNT3* in CAPAN-2 cells was much higher than that in various other cancer cells.

**Figure 3:**
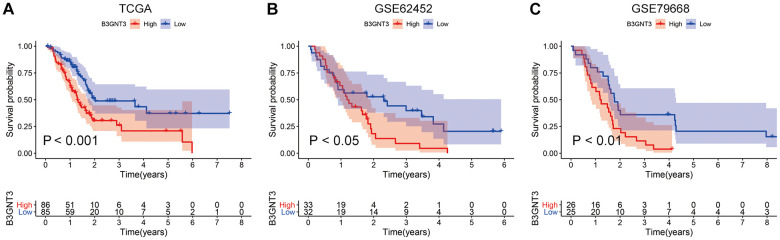
**KM survival analysis for *B3GNT3* through the TCGA, GSE62425, and GSE79668 datasets.** (**A**–**C**) High expression of *B3GNT3* was significantly associated with poor survival in PC (P value < 0.05). PC, pancreatic cancer; KM, Kaplan-Meier.

*B3GNT3* was overexpressed in tumor tissues (*P* < 0.0001) according to GSE62452 dataset and GSE60979 dataset (Figure 1A, 1B). The Oncomine database (https://www.oncomine.org/resource/main.html) contained seven studies about PC, among which two studies revealed *B3GNT3* expression in tumor tissues was significantly higher compared with that in normal pancreatic tissues (*P* < 0.05) (Figure 1C, 1D). The results of the other five studies in the Oncomine database are also shown in Supplementary Figure 1 (*P* > 0.05). Moreover, the overexpression of *B3GNT3* in PC was also observed in the Gene Expression Profiling Interactive Analysis (GEPIA) database (https://gepia.cancer-pku.cn/) (Figure 1E). Moreover, in GSE28735 dataset, *B3GNT3* expression in tumor tissues was significantly upregulated compared with that in the adjacent non-tumor tissues (*P* < 0.0001) (Figure 1F). The human protein atlas (HPA) database (http://proteinatlas.org/) demonstrated that the protein expression of *B3GNT3* was notably upregulated in tumor tissues (Figure 2A, 2B), while the expression of *B3GNT3* in CAPAN-2 cells was obviously higher than that in various other cancer cells (Figure 2C). In addition, *B3GNT3* expression was mainly located in the cytoplasm of PC cells (Figure 2B). Of note, KM survival analysis according to the TCGA PC dataset (cutoff point = 41.5), GSE62452 dataset (cutoff point = 4.70), and GSE79668 dataset (cutoff point = 2860) demonstrated that patients with lower levels of *B3GNT3* expression had a superior OS than those with higher levels of *B3GNT3* expression (*P* < 0.05) (Figure 3A–3C). Taken together, our study suggests *B3GNT3* as a critical but unfavorable prognostic factor for PC.

### *B3GNT3* expression and the correlation with clinicopathological characteristics

**Figure 4:**
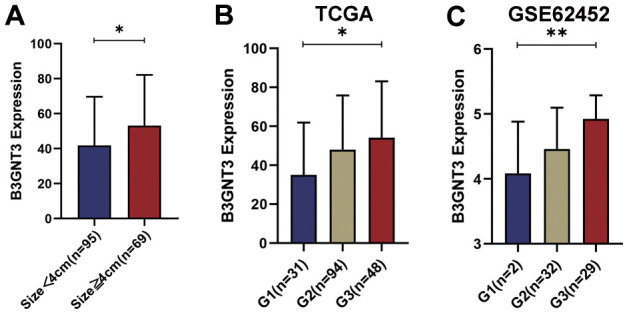
**The different *B3GNT3* expressions based on clinicopathological characteristics in the boxplot.** Boxplots showing the expression differences of *B3GNT3* according to tumor size (**A**) and tumor histologic grade (**B**, **C**). PC, pancreatic cancer; G1: grade 1; G2: grade 2; G3: grade 3. (*P value < 0.05; **P value < 0.01;***P value < 0.001; ****P value < 0.0001).

The correlation between the expression of *B3GNT3* and clinicopathological characteristics in the TCGA PC dataset and GSE62452 dataset are shown in Tables 1, 2. *B3GNT3* expression was significantly correlated with tumor size (*P* = 0.017) (Table 1) and histologic grade (*P* = 0.001) (Table 2). Patients with larger tumors (≥ 4 cm) and higher histologic grades had higher levels of *B3GNT3* expression (*P* < 0.05) (Figure 4). These results further suggest that *B3GNT3* overexpression may promote tumor progression in PC.

**Table 1:** Correlation between *B3GNT3* and clinicopathological characteristics in PC.

**Parameters**	**B3GNT3 expression**			**P value**
		**Low (n=88)**	**High (n=89)**	
**Age**	≤60	28 (31.8%)	30 (33.7%)	0.789
	>60	60 (68.2%)	59 (66.3%)	
**Gender**	Female	45 (51.1%)	35 (49.3%)	0.114
	Male	43 (48.9%)	54 (60.7%)	
**AJCC stage**	I	14 (15.9%)	7 (7.9%)	0.376
	II	69 (78.4%)	77 (86.5%)	
	III	2 (2.3%)	1 (1.1%)	
	IV	2 (2.3%)	2 (2.2%)	
	Unknown	1 (1.1%)	2 (2.2%)	
**Histologic grade**	G1	20 (22.7%)	11 (12.4%)	0.134
	G2	45 (51.1%)	49 (55.1%)	
	G3	21 (23.9%)	27 (30.3%)	
	G4	2 (2.3%)	0	
	Unknown	0	2 (2.2%)	
**Recurrence**	No	38 (43.2%)	33 (37.1%)	0.407
	Yes	50 (56.8%)	56 (62.9%)	
**Alcohol history**	No	32 (36.3%)	32 (36.0%)	0.852
	Yes	49 (55.7%)	52 (58.4%)	
	Unknown	7 (8.0%)	5 (5.6%)	
**Diabetes history**	No	51 (58.0%)	57 (64.0%)	0.566
	Yes	20 (22.7%)	18 (20.2%)	
	Unknown	17 (19.3%)	14 (15.7%)	
**Tumor size**	<4	56 (63.6%)	39 (43.8%)	0.017
	≥4	27 (30.7%)	42 (47.2%)	
	Unknown	5 (5.7%)	8 (9.0%)	
**Tumor site**	Head	72 (81.8%)	66 (74.2%)	0.112
	Body and Tail	10 (11.4%)	18 (20.2%)	
	Unknown	6 (6.8%)	5 (5.6%)	

Statistical significance was calculated by the χ2 test or the Fisher’s extract test. Bold values indicate *P* < 0.05 was considered statistically significant. G1: grade1; G2: grade2; G3: grade3.

**Table 2:** Correlation between *B3GNT3* and clinicopathological characteristics in PC in GSE62452 dataset.

**Parameters**	**B3GNT3 expression**			**P value**
		**Low (n=32)**	**High (n=33)**	
**AJCC stage**	I	1 (3.1%)	3 (9.1%)	0.146
	II	26 (81.3%)	18 (54.5%)	
	III	3 (9.4%)	8 (24.2%)	
	IV	2 (6.3%)	4 (12.1%)	
**Histologic grade**	G1	2 (6.3%)	0	**0.001**
	G2	21 (65.6%)	11 (33.3%)	
	G3	7 (21.9%)	22 (66.7%)	
	G4	1 (3.1%)	0	
	Unknown	1 (3.1%)	0	

Statistical significance was calculated by the χ2 test or the Fisher’s extract test. Bold values indicate *P* < 0.05 was considered statistically significant. G1: grade1; G2: grade2; G3: grade3.

### *B3GNT3* expression and correlation with somatic mutation

**Figure 5:**
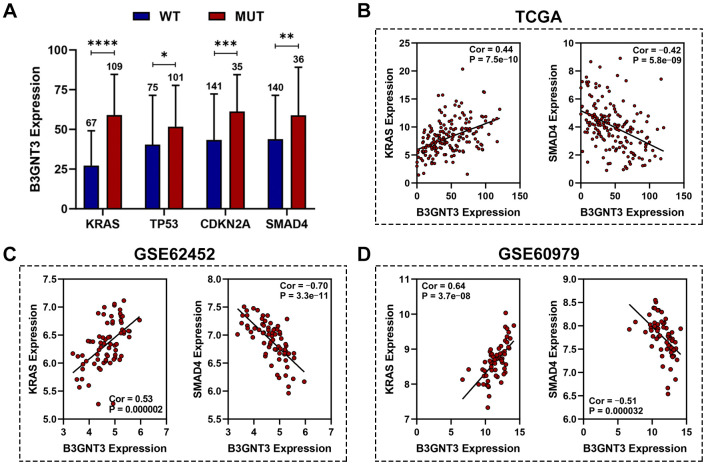
**Association between *B3GNT3* expression and somatic mutations.** (**A**) *KRAS*, *TP53*, *CDKN2A*, and *SMAD4* mutation status were significantly associated with higher expression of *B3GNT3*. (**B**–**D**) Correlation analysis of *KRAS*, *SMAD4*, and *B3GNT3*. PC, pancreatic cancer; TCGA, the Cancer Genome Atlas; WT: wild type; MUT: mutation. (*P value < 0.05; **P value < 0.01;***P value < 0.001; ****P value < 0.0001).

Previous studies have demonstrated that *KRAS*, *TP53*, *SMAD4*, and *CDKN2A* mutations are four of the most common genetic hallmarks of PC [20–22]. Our study demonstrated that *KRAS*, *TP53*, *CDKN2A*, and *SMAD4* mutation were significantly associated with higher levels of *B3GNT3* expression (Figure 5A). Pearson correlation analyses (|Cor| > 0.3 and *P* < 0.05) were conducted in the TCGA PC, GSE62452, GSE60979, and GSE79668 datasets. We excluded the GSE28735 dataset from the correlation analysis because GSE28735 was actually part of the GSE62452 dataset. We found that *B3GNT3* expression in the TCGA PC dataset was positively correlated with *KRAS* expression (Cor = 0.44, *P* < 0.0001), while it was negatively correlated with *SMAD4* expression (Cor = ‒0.42, *P* < 0.0001) (Figure 5B). Similar results were also obtained in GSE62452 (Cor = 0.53, *P* < 0.0001 for *KRAS*; Cor = ‒0.70, *P* < 0.0001 for *SMAD4*) and GSE60979 datasets (Cor = 0.64, *P* < 0.0001 for *KRAS*; Cor = ‒0.51, *P* < 0.0001 for *SMAD4*) (Figure 5C, 5D). In addition, the associations among *B3GNT3*, *KRAS*, and *SMAD4* in PC were also validated using the cBioportal database, the results of which were consistent with our findings (Supplementary Figure 2A, 2B). These findings suggest that *B3GNT3* overexpression is correlated with the mutation and expression patterns of driver genes, especially for *KRAS* and *SMAD4*.

### Functional enrichment analysis for *B3GNT3*

**Figure 6:**
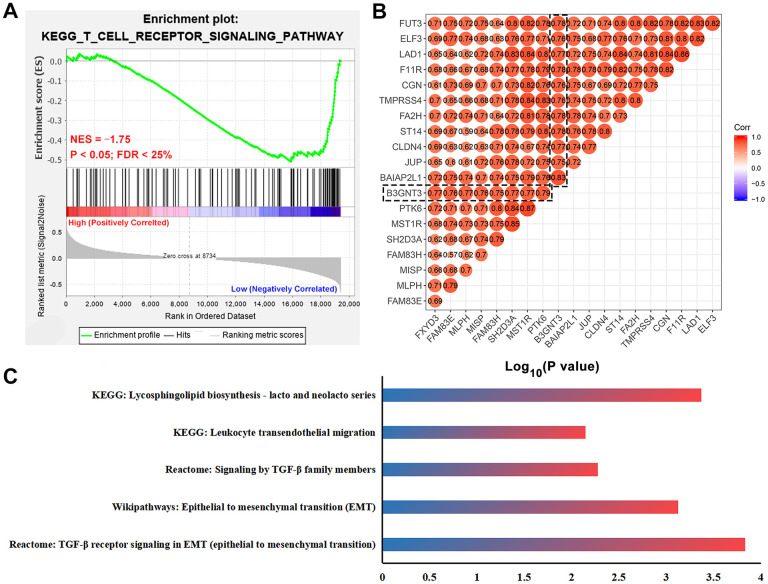
**Functional enrichment analysis for *B3GNT3*.** (**A**) GSEA revealed that T cell receptor signaling pathway related gene set (NES = -1.75, P < 0.05, and FDR < 25%) was enriched in the low *B3GNT3* expression group. (**B**) Co-expression analysis for *B3GNT3* in the TCGA PC dataset using Pearson correlation coefficients (Pearson correlated coefficient (Cor) > 0.75 or < -0.75, P value < 0.05). (**C**) Pathway enrichment analysis for *B3GNT3*. GSEA: gene set enrichment analysis; EMT: epithelial to mesenchymal transition; TCGA, the Cancer Genome Atlas; PC: pancreatic cancer.

First, we used gene set enrichment analysis (GSEA) to determine whether these pathways and their associated genes differ between the high *B3GNT3* and low *B3GNT3* expression groups. We found that the T cell receptor signaling pathway-related gene set (NES = ‒1.75, P < 0.05, FDR < 25%) was enriched in the low *B3GNT3* expression group (Figure 6A). Then, we conducted co-expression analysis (|Pearson correlation coefficient| > 0.75, *P* < 0.05) for *B3GNT3* based on the TCGA dataset. As a result, 19 co-expressed genes for B3GNT3 were subsequently uploaded into ConsensuspathDB for pathway enrichment analysis (*P* < 0.05) (Figure 6B) [23]. *B3GNT3* was found to be potentially involved in glycosphingolipid biosynthesis, leukocyte transendothelial migration, TGF-β signaling pathway, EMT, and TGF-β signaling pathway in EMT (Figure 6C). These findings indicate that *B3GNT3* overexpression provides the necessary support for tumor growth and immune regulation of PC.

### *B3GNT3* knockdown inhibits the proliferation, invasion, and EMT of PC cells

**Figure 7:**
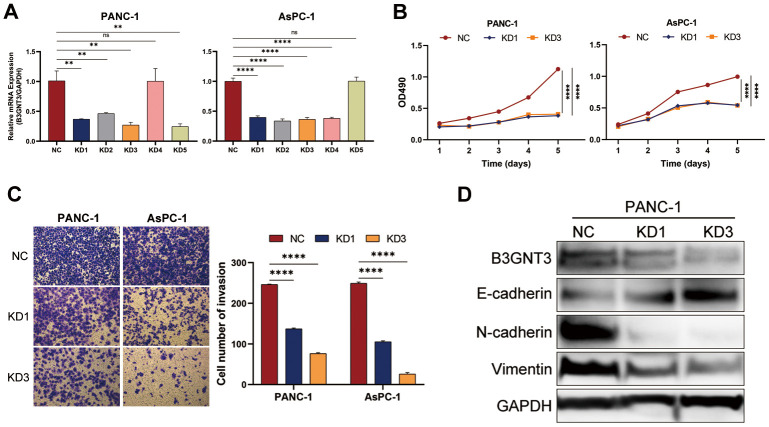
**The knockdown of *B3GNT3* suppresses the proliferation, invasion, and EMT of PC cells.** (**A**) RT-PCR analysis validated the knockdown of *B3GNT3* in PANC-1 and AsPC-1 cells transfected with sh-*B3GNT3*. (**B**) Assessment of cell proliferation using the MTT assay. (**C**) Transwell assay was performed to determine the invasive capacity of PANC-1 and AsPC-1 cells transfected with sh-*B3GNT3*. (**D**) WB analysis to investigate the association between *B3GNT3* and EMT in PC. PC: pancreatic cancer; EMT: epithelia-mesenchymal transition; WB: western blot. (*p<0.05; **p<0.01;***p<0.001; ****p<0.0001).

To further evaluate the functional role of *B3GNT3* in tumor cell proliferation and invasion, we transfected PANC-1 and AsPC-1 cells with sh-*B3GNT3*. Abrogated levels of *B3GNT3* in these two cells were validated using RT-qPCR analysis (*P* < 0.001) (Figure 7A). Then, the subclones KD1 and KD3 were selected for further experimental studies. The MTT assay revealed that cell proliferation was significantly inhibited in *B3GNT3*-depleted PANC-1 and AsPC-1 cells (*P* < 0.0001) (Figure 7B). Moreover, the transwell assay demonstrated that the number of invading cells was significantly decreased in the sh-*B3GNT3* groups (KD1 and KD3) compared with those in the NC group (*P* < 0.0001) (Figure 7C). Western blot assay demonstrated that the expression of E-cadherin was increased, while the expression of N-cadherin and vimentin protein was decreased in the sh-*B3GNT3* groups (KD1 and KD3) (Figure 7D). These findings suggest that the *B3GNT3* knockdown significantly inhibits the proliferation, invasion, and EMT of PC cells.

### *B3GNT3* overexpression inhibits the infiltration of CD8^+^ T cell in PC

**Figure 8:**
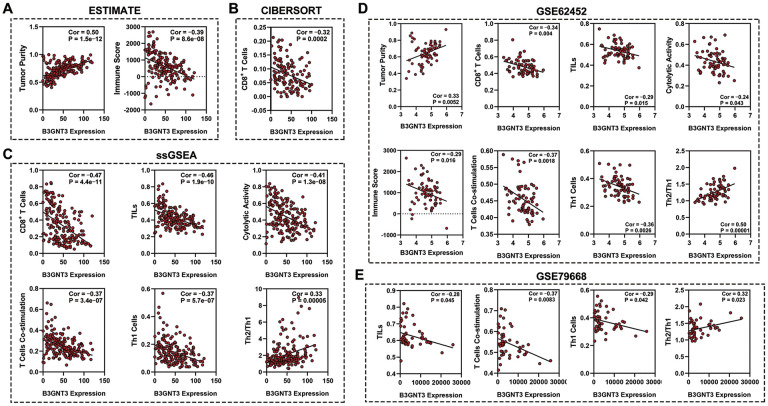
**Association between B3GTN3 expression and the immune infiltration within tumor.** (**A**) ESTIMATE algorithm to analyze the correlation between *B3GNT3* expression and immune infiltration in PC. (**B**) CIBERSORT algorithm to analyze the correlation between *B3GNT3* expression and immune infiltration in PC. (**C**) ssGSEA analysis to analyze the correlation between *B3GNT3* expression and the immune infiltration in PC. (**D**) ESTIMATE algorithm and ssGSEA to analyze the correlation between *B3GNT3* expression and immune infiltration within tumors in GSE62452 dataset. (**E**) ssGSEA analysis to analyze the correlation between *B3GNT3* expression and immune infiltration within tumors in GSE79668 dataset. TCGA, the Cancer Genome Atlas; PC: pancreatic cancer; ssGSEA: single-sample gene set enrichment analysis; TILs: tumor-infiltrating lymphocytes; Th1 cells: Type-1 T helper cells; Th2/Th1: Type-2 T helper cells/ Type-1 T helper cells.

**Figure 9:**
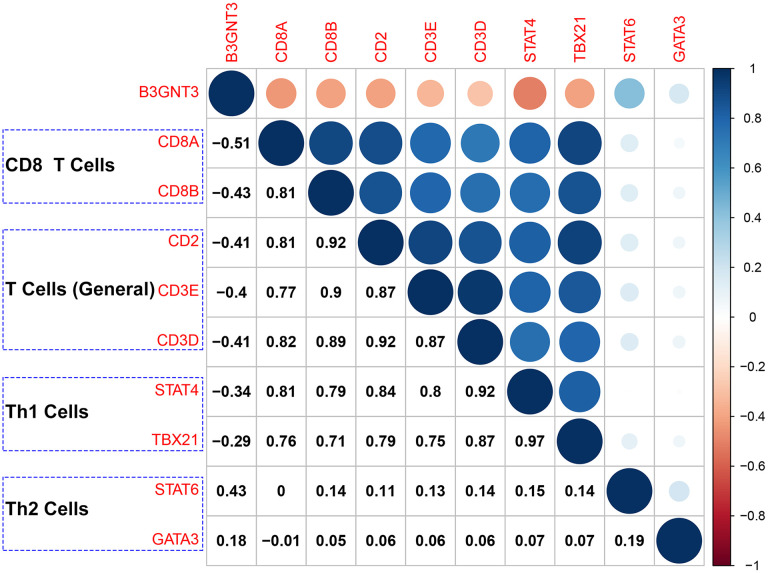
**Correlation analysis of *B3GNT3* expression and the expression of marker genes of CD8^+^ T cells (*CD8A* and *CD8B*), T cells (general) (*CD2*, *CD3D*, and *CD3E*), Th1 cells (*STAT4* and *TBX21*), and Th2 cells (*STAT6* and *GATA3*).** Th1 cells: Type-1 T helper cells; Th2 cells: Type-2 T helper cells.

The ESTIMATE algorithm showed that higher *B3GNT3* expression was significantly correlated with higher tumor purity (Cor = 0.50, *P* < 0.0001), but lower immune score (Cor = ‒0.39, *P* < 0.0001) (Figure 8A), a finding that was also observed in the GSE62452 dataset (Cor = 0.33, *P* = 0.0052 for tumor purity; Cor = ‒0.29, *P* = 0.016 for immune score) (Figure 8D). Using the CIBERSORT algorithm, we found that *B3GNT3* overexpression significantly correlated with lower CD8^+^ T cells infiltration within the TME of PC (Figure 8B). In the TCGA PC dataset, similarly, ssGSEA analysis indicated that *B3GNT3* overexpression was notably correlated with lower infiltrations of CD8^+^ T cells (Cor = ‒0.47, *P* < 0.0001), TILs (Cor = ‒0.46, *P* < 0.0001), cytolytic activity (Cor = ‒0.41, *P* < 0.0001), T cell co-stimulation (Cor = ‒0.37, *P* < 0.0001), and Th1 cells (Cor = ‒0.37, *P* < 0.0001), but higher Th2/Th1 (Cor = 0.33, *P* < 0.0001) (Figure 8C), which was entirely consistent with those we observed in GSE62452 dataset (Cor = ‒0.34, *P* = 0.004 for CD8^+^ T cells; Cor = ‒0.29, *P* = 0.015 for TILs; Cor = ‒0.24, *P* = 0.043 for cytolytic activity; Cor = ‒0.37, *P* = 0.0018 for T cell co-stimulation; Cor = ‒0.36, *P* = 0.0026 for Th1 cells; Cor = 0.50, *P* < 0.0001 for Th2/Th1) (Figure 8D). Furthermore, similar results were also observed in the GSE79668 dataset, which showed that *B3GNT3* overexpression was significantly correlated with a low infiltration of TILs (Cor = ‒0.28, *P* = 0.045), T cell co-stimulation (Cor = ‒0.37, *P* = 0.0083), and Th1 cells (Cor = ‒0.29, *P* = 0.042), whereas it was correlated with high Th2/Th1 (Cor = 0.32, *P* = 0.023) (Figure 8E). Furthermore, *B3GNT3* expression was also negatively correlated with gene markers from CD8^+^ T cells (*CD8A* and *CD8B*), T cells (general) (*CD3D*, *CD3E*, and *CD2*), and Th1 cells (*STAT1* and *TBX21*), but positively correlated with gene markers from Th2 cells (*STAT6* and *GATA3*) (Figure 9). Similar results were observed in the GEPIA database (Table 3).

**Table 3:** Correlation analysis between *B3GNT3* and immune cells marker in GEPIA.

**Immune cells**	**Gene markers**	**Tumor**		**Normal**	
		**Cor**	**P value**	**Cor**	**P value**
**CD8**+ T Cells	CD8A	-0.36	**8.70E-07**	0.088	0.25
	CD8B	-0.38	**1.50E-07**	0.14	0.072
**T Cell (General)**	CD2	-0.38	**1.30E-07**	0.22	**0.0044**
	CD3D	-0.29	**8.00E-05**	0.18	**0.022**
	CD3E	-0.33	**6.40E-06**	0.15	0.054
**Th1 Cells**	STAT4	-0.45	**3.10E-10**	0.19	**0.011**
	T-bet (TBX21)	-0.38	**1.30E-07**	0.0036	0.96
**Th2 Cells**	STAT6	0.48	**9.40E-12**	0.35	**2.00E-06**
	GATA3	0.25	**6.50E-04**	0.29	**1.60E-04**

Bold values indicate P < 0.05 were considered statistically significant.

**Figure 10:**
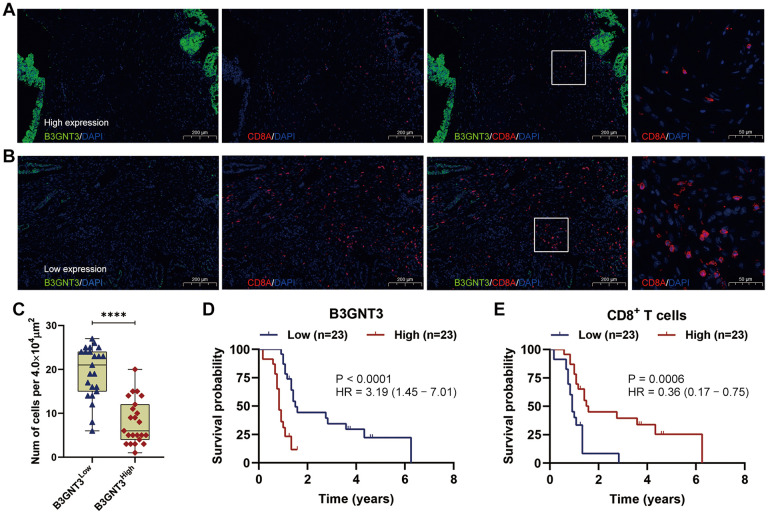
**Multi-color immunofluorescence to investigate the relationship between *B3GNT3* expression and CD8^+^ T cells infiltration in PC.** (**A**) High *B3GNT3* expression in multi-color immunofluorescence. (**B**) Low *B3GNT3* expression in multi-color immunofluorescence. (**C**) High *B3GNT3* expression group displayed a significant increased CD8^+^ T cells infiltration compared to low *B3GNT3* expression group. (**D**) Patients with higher *B3GNT3* expression had significant shorter OS that those with lower *B3GNT3* expression. (**E**) Patients with higher CD8^+^ T cells infiltration had superior OS than those with lower CD8^+^ T cells infiltration.

To validate our findings from the TCGA and GEO datasets, we investigated the correlation between the expression pattern of *B3GNT3* and the infiltration of CD8^+^ T cell by multi-color immunofluorescence in an independent dataset of 50 PC cases. Cases with less than 5% tumor or loss of tissues were excluded for the quantification of B3GNT3. Representative tumor cores were available from 46 patients. The high *B3GNT3* expression group displayed a significantly increased CD8^+^ T cell infiltration compared to the low *B3GNT3* expression group (Figure 10A–10C). Moreover, we further proved that *B3GNT3* overexpression positively correlated with a worse OS in PC patients, while a higher CD8+ T cells infiltration in TME positively correlated with a better OS of PC patients (Figure 10D, 10E). Taken together, these findings suggest that *B3GNT3* overexpression may inhibit TILs in PC, especially CD8^+^ T cells.

## DISCUSSION

*B3GNT3* has been demonstrated to be an oncogene in several cancers (e.g. lung cancer, pancreatic cancer, and breast cancer), and participates in lymphocyte trafficking and migration [13, 15, 16]. However, immune-related functional analyses of *B3GNT3* in cancer are currently limited to triple-negative breast cancer and non-Hodgkin lymphoma [14, 16]. In this study, we aimed to delineate the biological role of *B3GNT3* during pancreatic tumorigenesis and tumor immunity. Consistent with previous oncologic studies about *B3GNT3*, multiple PC databases indicated that *B3GNT3* was notably overexpressed in tumor tissues [12, 16, 24], and positively correlated with tumor size, histologic grade, and poor survival of patients with PC, which indicates that *B3GNT3* may be an unfavorable prognostic biomarker for PC. Based on these initial findings, we further explored the potential biological role of *B3GNT3* during tumor growth and in the regulation of immune infiltration in PC.

Through GSEA, we found that tumors with low levels of *B3GNT3* expression significantly enriched the T cell receptor signaling pathway-related gene set. Moreover, pathway enrichment analysis revealed that *B3GNT3* plays a role in glycosphingolipid biosynthesis, leukocyte transendothelial migration, TGF-β signaling pathway, and EMT induced by TGF-β. Our schema was further supported by the fact that *B3GNT3* knockdown significantly inhibited cell proliferation and invasion in PC cells. Importantly, the downregulation of *B3GNT3* impaired the process of EMT in PC. These findings suggest that *B3GNT3* overexpression in PC enhances the process of EMT in PC, thereby promoting tumor cell proliferation and invasion.

Previous studies have reported that TGF-β maintains tissue hemostasis and blocks cell cycle progression, thereby suppressing tumor formation [25, 26]. However, due to the inactivation of the TGF-β signaling mediator, *SMAD4,* the tumor suppressive role of TGF-β is always impaired in PC [27]. In the present study, *B3GNT3* was markedly upregulated by *SMAD4* mutations in PC and negatively associated with *SMAD4* expression. Thus, we propose that *B3GNT3* overexpression induces *SMAD4* inactivation and promotes tumor growth and metastasis through TGF-β signaling pathway. However, further experimental studies are needed to demonstrate our proposed *B3GNT3*-*SMAD4*-TGF-β pathway hypothesis.

Our study also identified the novel role of *B3GNT3* in tumor immunity of PC. The ESTIMATE algorithm showed that high levels of *B3GNT3* expression were associated with increased tumor purity, but decreased immune score, suggesting that *B3GNT3* overexpression may promote immunosuppression in PC. Both the CIBERSORT algorithm and ssGSEA demonstrated that *B3GNT3* overexpression was significantly associated with a low infiltration of TILs (e.g. CD8^+^ T cells). Moreover, *B3GNT3* was negatively correlated with CD8^+^ T cell markers, *CD8A* and *CD8B*, and general T cell markers, *CD2*, *CD3D*, and *CD3E*. Notably, multi-color immunofluorescence demonstrated that the high *B3GNT3* expression group displayed a significantly increased CD8^+^ T cell infiltration compared to the low *B3GNT3* expression group. These findings suggest that *B3GNT3* overexpression may reduce CD8^+^ T cells infiltration in PC. A previous study reported that *B3GNT3* overexpression impaired cytotoxic T cell-mediated anti-tumor immunity in triple-negative breast cancer [16]. However, this study is the first to demonstrate that *B3GNT3* overexpression inhibits the infiltration of CD8^+^ T cells and cytolytic activity in PC.

Previous studies have reported that *KRAS*-mutated cancer cells help to build an immunosuppressive environment by regulating immune cells behavior in PC [28]. For example, *KRAS*-mutated cancer cells could induce myeloid-derived suppressor cells trafficking by secreting granulocyte macrophages and colony-stimulating factor (GM-CSF), which inhibits the behavior of CD8^+^ T cells [29]. *KRAS* activation was also reported to impede the antigen presentation pathway, thereby allowing the evasion of CD8^+^ T cells [30]. It has been reported that *KRAS* mutations in PC upregulate TGF-β levels, thereby downregulating CD8^+^ T cell infiltration, switching Th1/Th2, and hampering T cell co-stimulation, and subsequently contributes to immunosuppression in the TME [28, 31, 32]. Increasing studies show that the excessive TGF-β expression in tumors is highly related to the anti-tumor effect of CD8^+^ T cells [33]. Furthermore, Leung et al. reported that *SMAD4* inactivation promotes *KRAS*-mediated malignant transformation, thereby promoting tumor progression in PC [34]. Our study demonstrates that high levels of *B3GNT3* expression are associated with *KRAS* mutation and high *KRAS* expression. Based on our results, we propose that *B3GNT3* overexpression, *SMAD4* inactivation, and *KRAS* activation may interact to promote tumor growth and inhibit the infiltration of CD8^+^ T cells in PC.

In the present study, *B3GNT3* overexpression was correlated with higher Th2/Th1 but a lower infiltration of Th1 cells (*STAT1* and *TBX21*). Th2/Th1 is widely considered an independent unfavorable prognostic factor in post-surgery patients with PC [35–37]. Furthermore, Th2/Th1 has been reported to be inversely correlated with the presence of CD8^+^ T cells in PC [38, 39]. Th1 cells are crucial for the effective anti-cancer function of the immune system [36, 37]. Th2 cells drive tumor-promoting inflammation, leading to poor survival outcomes of PC [39]. The infiltration of CD8^+^ T cells in patients with PC is inhibited or impaired when Th2 dominates the TME [40]. Moreover, DeNardo et al. demonstrated that Th2 cells can produce *IL4*, *IL10*, and *IL13*, which impair the cytolytic activity of CD8^+^ T cells [41]. These results imply that *B3GNT3* overexpression in PC switched Th1/Th2, which induced the domination of Th2 cells in the TME of PC, leading to an immunosuppressive TME with a limited infiltration of CD8^+^ T cells. Thus, targeting *B3GNT3* may recover CD8^+^ T cell infiltration within the TME of PC, which could be a novel therapeutic strategy for PC.

The current study demonstrated that *B3GNT3* overexpression may promote TGF-β-induced EMT in PC. Previously, several studies have suggested that TGF-β promotes EMT, which induces immune evasion or suppression in the TME [42, 43]. In their review, Jiang et al. found that the EMT played a pivotal role in tumor immunosuppression and immune evasion [44]. Besides, the process of EMT promotes therapeutic resistance to ICIs [45, 46]. Li et al. suggested targeting *B3GNT3* as a novel strategy to enhance immune checkpoint therapies [16]. It has also been reported that inhibiting TGF-β signaling pathway could improve the efficacy of ICIs [33]. Similarly, we proposed that blocking EMT or TGF-β by targeting *B3GNT3* may be an effective therapeutic direction to enhance current immune-based therapies.

As far as we know, this study is the first to describe an association between the expression of *B3GNT3* and CD8^+^ T cell infiltration in PC. However, there are some limitations. First, the outcome of this studies was somehow influenced by the quality of data from public databases. Second, the biological role of *B3GNT3* in pancreatic carcinogenesis and the modulation of tumor immunity has only been preliminarily validated *in vitro* and through multi-color immunofluorescence. Future studies should be conducted to determine how *B3GNT3* induces immune suppression through the EMT or TGF-β signaling pathways in PC.

In conclusion, our findings indicate that *B3GNT3* overexpression promoted tumor progression and was notably associated with a decreased infiltration and cytolytic activity of CD8^+^ T cells, but increased Th2/Th1. Thus, we established *B3GNT3* as a novel prognostic biomarker, inhibiting CD8^+^ T cell infiltration in PC, and a promising therapeutic target in PC.

## MATERIALS AND METHODS

### Acquisition of data

We obtained the RNA-sequencing data and corresponding clinical data of PC patients from The Cancer Genome Atlas (TCGA, https://cancergenome.nih.gov/) in February 2020. Mutation information for *KRAS*, *TP53*, *SMAD4*, and *CDKN2A* in the TCGG PC dataset were downloaded through the cBioportal database (http://www.cbioportal.org/). Of the 177 patients in TCGA PC dataset, 171 had an OS > 1 month. In addition, the relevant PC datasets were retrieved from the Gene Expression Omnibus (GEO) (http://www.ncbi.nlm.nih.gov/geo/) using the following search words: “pancreatic ductal adenocarcinoma” and “pancreatic cancer”. The exclusion criteria included: (1) studies involving only blood samples; (2) studies with fewer than 30 PC samples; (3) studies involving only PC cell lines or xenografts. Finally, four GEO datasets, including GSE62452 [GPL6244 (HuGene-1_0-st) Affymetrix Human Gene 1.0 ST Array (transcript (gene) version); 61 non-tumor samples and 69 pancreatic tumor samples], GSE60979 [GPL14550 SurePrint G3 Human GE 8x60K Microarray; 12 non-tumor samples and 49 pancreatic cancer samples], GSE28735 [GPL6244 (HuGene-1_0-st) Affymetrix Human Gene 1.0 ST Array (transcript (gene) version); 45 pairs of pancreatic tumor and adjacent non-tumor tissues], and GSE79668 (GpL11154 Illumina HiSeq 2000 (Homo sapiens); 51 pancreatic tumor samples], were selected for further analysis [47]. All datasets involved in this study are publicly available. Thus, local ethics approval was not required. A flowchart of our study is provided in Supplementary Figure 3.

### Differential expression analysis for B3GNT3

Multiple datasets were utilized to evaluate *B3GNT3* expression in PC. First, differential expression analysis for *B3GNT3* was conducted using GSE62452 dataset and GSE60979 dataset. Then, the Oncomine database and GEPIA database were further used to analyze B3GNT3 expression in PC. Paired differential expression analysis for *B3GNT3* was also conducted using GSE28735 dataset. Moreover, the HPA database was used to further validate *B3GNT3* protein expression in PC samples and normal pancreatic tissues, and *B3GNT3* expression in cancer cell lines.

### Kaplan-Meier survival analysis

GSE62452, GSE79668 and TCGA datasets were used in survival analysis to assess the prognostic value of B3GNT3 expression in PC. We divided patients in these three PC datasets, respectively, into high- and low-*B3GNT3* expression groups, according to the medium cutoff of *B3GNT3* expression. KM survival curves were conducted using the “survminer” package in R.

### Functional enrichment analysis

First, we performed GSEA to evaluate the differences in possible biological pathways between the high- and low-*B3GNT3* expression groups in the TCGA PC dataset. An annotated gene set c2.cp.kegg.6.2.symbols.gmt obtained from the Molecular Signatures Database (MSigDB) was used as the reference gene set. Genes significantly related to *B3GNT3* in the TCGA PC dataset were sniffed out with |Pearson correlation coefficient (Cor)| >0.75 and *P* < 0.05. These co-expressed genes of *B3GNT3* were subsequently uploaded into ConsensuspathDB (http://cpdb.molgen.mpg.de/) for functional enrichment analysis, with *P* < 0.01 considered significant [23].

### Cell culture and transfection

We purchased Human PC cell lines, PANC-1 and Aspc-1 from the Cell Bank of the Chinese Academy of Science (Shanghai, China). Cells were subsequently cultured in RPMI-1640 medium (Invitrogen, Carlsbad, CA, USA) containing 10% fetal bovine serum (FBS) (HyClone, Logan, UT, USA) and 1% penicillin/streptomycin in a humidified 5% CO_2_ incubator at 37° C [48]. Cells were plated in 6-well plates at a density of 1 × 10^6^ cells per well before transfection [48]. Lentiviral small hairpin RNA (shRNA) targeting *B3GNT3* was synthesized and cloned into the GV248 vector (Genechem Co., Ltd, Shanghai, China). As previous studies introduced [48, 49], at 80% confluence, the *B3GNT3* lentiviruses (sh-*B3GNT3*) and the negative control shRNA (NC) were transfected into the cells using Lipofectamine 2000 (Invitrogen). At 48 h post-transfection, the cells were harvested for further analyses. The efficacy of sh-*B3GNT3* for each cell line was assessed using qRT-PCR. The PCR primers were as follows: *B3GNT3* forward, 5′-CAGCACGTTCAGAACTTCCTC-3′; *B3GNT3* reverse, 5′-GCGCACATAGTTGCTAGGGG-3′; *GAPDH* forward, 5′-TGACTTCAACAGCGACACCCA-3′; *GAPDH* reverse, 5′-CACCCTGTTGCTGTAGCCAAA-3′.

### MTT assay

The 3-(4,5-dimethylthiazal-2-yl)-2, 5-diphenyl-tetrazolium bromide (MTT) assay was used to measure PC cell proliferation. Cells were plated in 96-well plates at 2,000 cells/well and cultured for 1-5 days. At the indicated periods, 20 μL of MTT (5 mg/mL) (Sigma-Aldrich, St. Louis, MO, USA) solution was added to each well and further incubated for 4 h at 37° C [50]. Then, 100 μL of DMSO (Corning Inc., Corning, NY, USA) was added to each well to solubilize the formation product. Finally, a microplate reader (Bio-Tek Company, Winooski, VT, USA) was utilized to measure the absorbance at 490 nm, as previously described [48, 51].

### Transwell invasion assays

The cell invasion assay was conducted using Transwell Permeable Supports (Corning Inc., Corning, NY, USA), as previously described [48, 52]. Cells (1.0 × 10^5^) suspended in serum-free medium were plated in triplicate onto 8-μm Transwell filter inserts of 24-well plates pre-coated with Matrigel (10 mg/L) (BD Biosciences, San Jose, CA, USA). And 10% fetal bovine serum (FBS) was added into the lower chamber. After 18 h, the cells in the upper chamber were wiped with a cotton-tipped swab. Then, the chambers were fixed with 100% methanol and stained with 0.5% crystal violet for 20 min. Images of invaded cells were obtained through an inverted light microscope (Olympus Corp., Tokyo, Japan). Nine fields were observed at random in each group.

### Western blot analysis

Radio immunoprecipitation assay lysis buffer containing proteinase inhibitors (Beyotime, Jiangsu, China) was utilized to extract protein from cultured cells. Protein concentrations were determined using the BCA Protein Assay Kit (Pierce, Rockford, IL, USA). Protein samples (30 μg) from each group were resolved on 10% SDS-PAGE and transferred onto nitrocellulose membranes [51]. Then, the membranes were then incubated with anti-*B3GNT3* (NBP1-32539; Novus Biologicals), E-Cadherin (ab40772; Abcam), N-cadherin (ab76011; Abcam), vimentin (ab92547, Abcam), and anti-*GAPHD* (ab181602; Abcam). After washing, the membranes were subsequently incubated with secondary horseradish peroxidase-conjugated antibody (1:5000; Santa Cruz Biotechnology) for 1 h at room temperature. Protein-expressing signals were visualized using the Western Lightning Plus ECL kit (PerkinElmer, Waltham, MA, USA) and quantified by densitometry, as previously described [50].

### Immune infiltration analysis in PC through multiple datasets

As previously introduced [53], we were using the “estimate” package in R to perform the ESTIMATE algorithm in the TCGA PC dataset, which was based on ssGSEA to generate a tumor purity score and an immune score [18]. PC samples with high immune scores showed high level of immune cells infiltration within the TME. A higher tumor purity score indicates a lower immune infiltration in tumor tissues. The CIBERSORT algorithm is an analytic tool that estimates specific cell type frequencies using gene expression data. Thus, we used the CIBERSORT algorithm to estimate the fraction of 22 immune cell types in each tumor tissue in the TCGA PC dataset [17]. Next, using the “gsva” package in R, ssGSEA was conducted to calculate the enrichment scores of each immune-related term in the TCGA PC dataset [19]. The gene sets of the following 29 immune-related terms (CD8^+^ T cells, cytolytic activity, regulatory T cells (Treg), tumor-associated macrophages (TAMs), natural killer cells (NK cells), type-2 T helper cells (Th2 cells), type-1 T helper cells (Th1 cells), antigen-presenting cell (APC) co-stimulation, antigen-presenting cell (APC) co-inhibition, type-1 IFN response, major histocompatibility complex (MHC) class-1, parainflammation, plasmacytoid dendritic cells (pDCs), T cell co-stimulation, activated dendritic cells (aDCs), check-point, T cell co-inhibition, B cells, follicular helper T cells (Tfh), neutrophils, tumor-infiltrating lymphocyte (TIL), inflammation-promoting, type-1 IFN response, human leukocyte antigen (HLA), T helper cells, chemokine receptor (CCR), mast cells, dendritic cells, and immature dendritic cells (iDCs)) were obtained from a previous study [54]. Correlation analysis between *B3GNT3* expression and immune infiltration was conducted using Pearson correlation coefficients (|Cor| > 0.3 and *P* < 0.05) [55]. The GSE62452 and GSE79668 datasets were used to validate the immune infiltration landscape of PC using ESTIMATE and ssGSEA. Furthermore, we conducted correlation analysis between *B3GNT3* and specific immune cells markers to further confirm the association between *B3GNT3* and immune infiltration in PC.

### Tissue microarray (TMA) construction

A total of 50 samples of PC patients were collected after R0 resection in Guangdong Provincial People’s Hospital during 2013-2020. All PC patients were regularly followed up in our institution. The OS of these 50 patients was >1 month. Thereafter, a TMA was constructed for the 50 samples using 1.5 mm tissue cores. However, the final number of samples analyzed was lower due to the absence or limited tumor cells in some samples or unavoidable loss of tissue, as commonly occurs in TMA studies [56]. All tissues were approved by the Clinical Research Ethics Committee of Guangdong Provincial People’s Hospital. Informed consent was obtained from all participants.

### Multi-color immunofluorescence

We measured the expression level of *B3GNT3* using multi-color immunofluorescence in the TMA slides. *B3GNT3* (NBP1-32539; Novus Biologicals) and *CD8A* (ab17147; Abcam) were simultaneously stained in serial sections from the TMA blocks. In short, antigen retrieval was performed with citrate buffer pH 8.0 for 20 min at 97° C in a pressure-boiling container. Blocking was subsequently conducted using 0.3% bovine serum albumin in 0.05% Tween solution for 30 min [56], followed by incubation with primary antibodies at 4° C overnight. At last, fluorescein-labeled secondary antibodies were added for signaling detection at room temperature. Nuclei were detected with DAPI. The samples were photographed using a laser confocal microscope. The area quantification the FL method of Halo software v3.0.311.314 (Indica Labs, USA) was used to quantify the intensities of the *B3GNT3* fluorescent signals. Then, we divided the samples into high- and low-*B3GNT3* expression groups according to the medium cutoff of the *B3GNT3* fluorescent signal intensity. On each slide, three stromal areas of 4 × 10^4^ μm^2^ were randomly selected based on DAPI staining. Within each area, CD8^+^ T cells were counted manually, and the average CD8^+^ T cell counts within the three stromal areas (4 × 10^4^ μm^2^) were calculated for each slide. Lastly, we evaluated the differences in CD8^+^ T cell count (per 4 × 10^4^ μm^2^) between the high- and low-B3GNT3 expression groups.

### Statistical analysis

All statistical analyses were performed using SPSS 25.0 software, R 3.5.2 software (http://r-project.org/), and GraphPad Prism 8.0 software (GraphPad Software, Inc.). Group differences were analyzed by Wilcoxon test or Kruskal-wallis test, and expressed as the mean ± standard deviation (SD). Correlation analysis was performed with the Pearson correlation coefficient. *P* < 0.05 was considered statistically significant.

## Supplementary Material

Supplementary Figures
