# Supplementary material for: B3GNT3 overexpression promotes tumor progression and inhibits infiltration of CD8+ T cells in pancreatic cancer
Source: Aging (Albany NY). 2020 Dec 9;13(2):2310–29. doi: 10.18632/aging.202255 (PMC7880340; doi:10.18632/aging.202255)
Supplement: Supplementary Figures [file aging-13-202255-s001.pdf]

SUPPLEMENTARY FIGURES

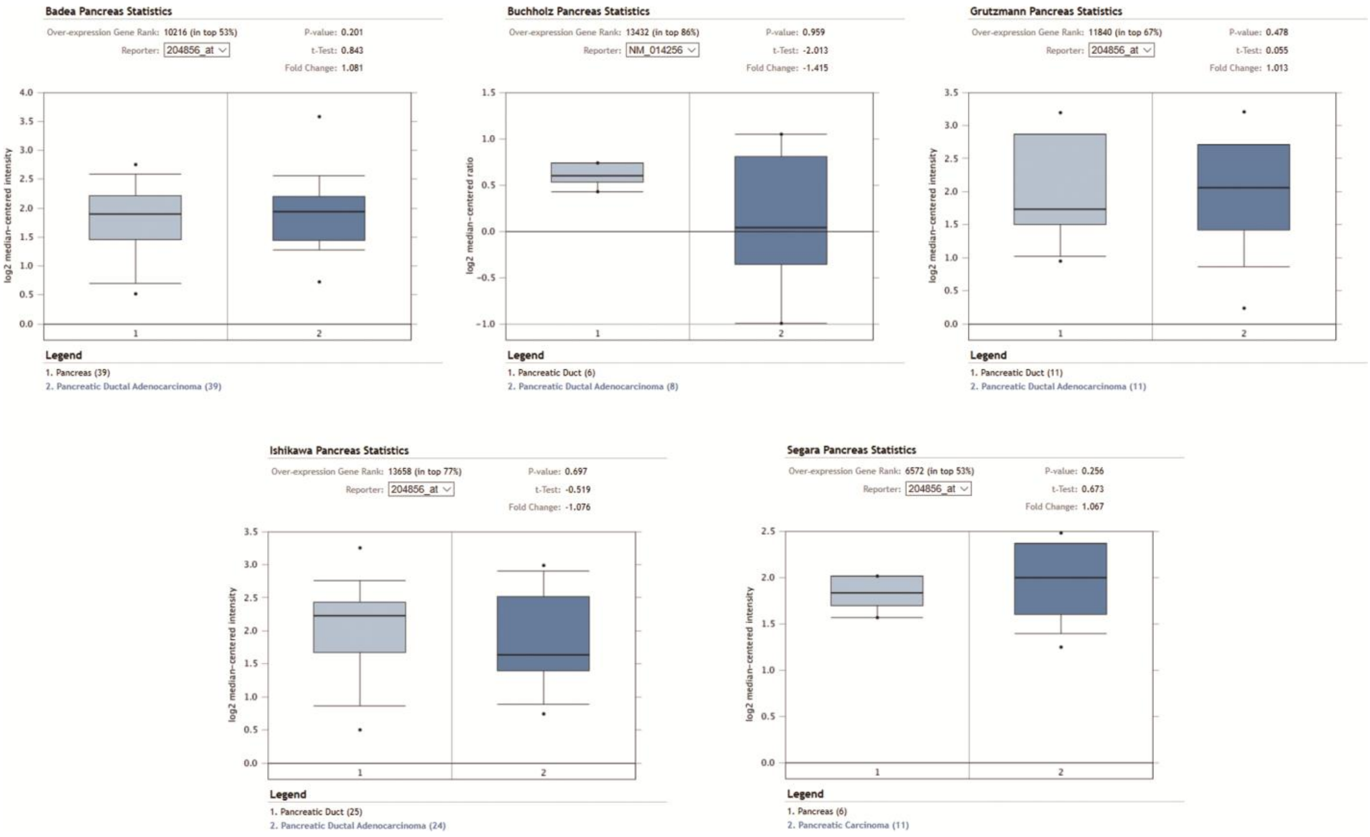

Supplementary Figure 1. ***B3GNT3*** **expression analysis based on the other five studies in the Oncomine database.**

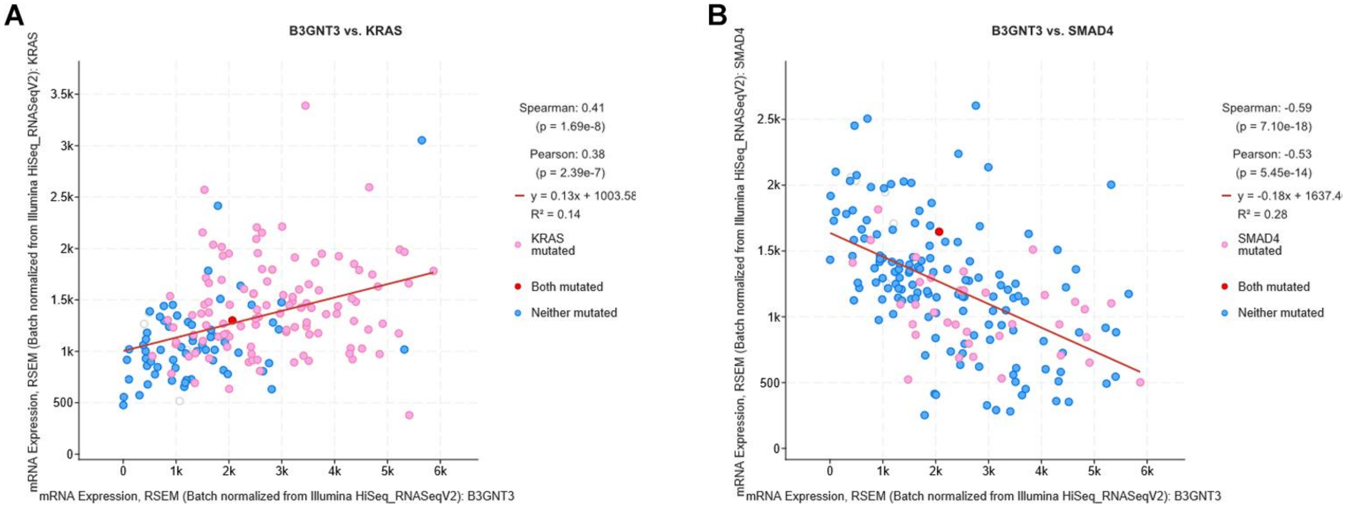

Supplementary Figure 2. Validation of the association among *B3GNT3*, *KRAS*, and *SMAD4* using cBioportal database. (**A**) *B3GNT3* expression was positively correlated to *KRAS* expression. (**B**) *B3GNT3* expression was negatively correlated to *SMAD4* expression.

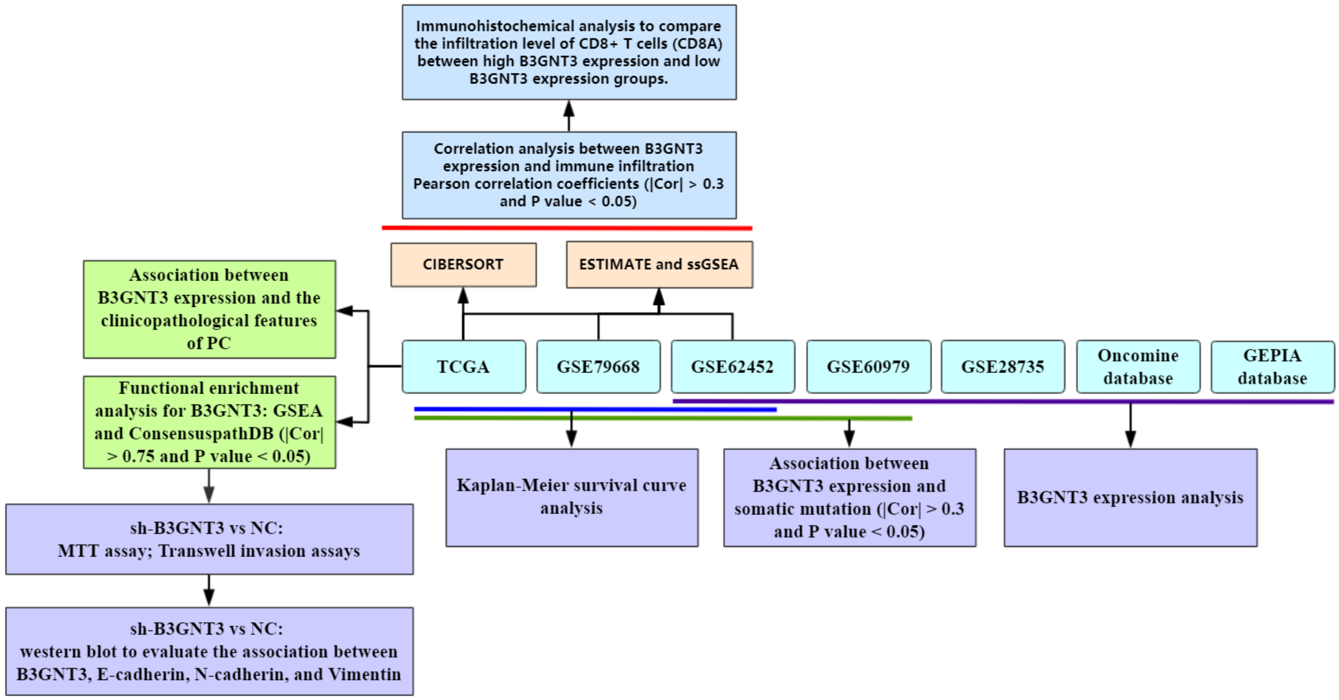

Supplementary Figure 3. Flowchart of the whole study.
